# Explainable machine learning-based identification of transcriptomic biomarkers in CD1c+ dendritic cells for non-infectious uveitis: an integrative analysis of bulk RNA-seq data

**DOI:** 10.3389/fbinf.2026.1861968

**Published:** 2026-06-11

**Authors:** Yelda Fırat

**Affiliations:** Department of Computer Engineering, Mudanya University, Bursa, Türkiye

**Keywords:** CD1c+ dendritic cells, explainable machine learning, non-infectious uveitis, SHAP analysis, transcriptomic biomarkers

## Abstract

**Introduction:**

Non-infectious uveitis (NIU) is a leading cause of intraocular inflammation, and the underlying immunological mechanisms remain incompletely understood. This study aims to classify NIU at the molecular level and interpret its biological mechanisms by applying machine learning and explainable artificial intelligence (XAI) methods to transcriptomic data from CD1c+ conventional dendritic cells type 2 (cDC2) isolated from the peripheral blood of NIU patients.

**Methods:**

Two independent cohorts (GSE194060 and GSE195501; n = 78) from the Gene Expression Omnibus (GEO) database were integrated using a strict, leakage-free pipeline. Batch correction and analysis of variance (ANOVA)-based feature selection, applied exclusively on the training set, identified the 20 most informative genes from 8,815 candidates. L2-regularized logistic regression, support vector machines, and random forest models were compared. The model’s biological basis was elucidated using Shapley Additive Explanations (SHAP) analysis and comprehensive pathway enrichment analyses.

**Results:**

The L2-regularized logistic regression model achieved the best performance with a nested cross-validation area under the curve (AUC) of 0.863, test AUC of 0.855, accuracy of 81.2%, recall of 90.9%, and F1-score of 0.870. Leave-One-Dataset-Out (LODO) validation supported cross-platform generalizability (mean AUC: 0.82). Permutation testing confirmed the model’s statistical significance (p = 0.005). SHAP analysis identified CD180 and TLR7 as the most important biomarkers. Pathway enrichment analysis revealed significant enrichment in chemokine receptor activity, MYD88-mediated signaling, Toll-like receptor cascades, and G protein-coupled receptor (GPCR) signaling pathways.

**Conclusion:**

These findings demonstrate the potential of XAI to elucidate disease mechanisms from transcriptomic data and present an interpretable 20-gene signature as a candidate diagnostic biomarker panel for non-infectious uveitis that warrants further clinical validation.

## 1 Introduction

Non-infectious uveitis (NIU) accounts for 10%–15% of preventable blindness in developed countries (Tsirouki et al., 2018; Gritz and Wong, 2004). Epidemiological studies have shown that NIU constitutes the vast majority of uveitis cases in developed countries, with an estimated annual incidence of 17–52 per 100,000 people and a prevalence of 38–714 per 100,000 people, placing a significant burden on healthcare systems (Joltikov and Lobo-Chan, 2021). The pathogenesis of NIU is complex, involving disruption of ocular immune privilege and an autoimmune or autoinflammatory response to ocular tissues (Lee et al., 2014; Forrester et al., 2018). Experimental models and clinical observations have shown that autoimmune and inflammatory uveitis arises without a known infectious trigger and is often associated with immunological responses to unique retinal proteins (Caspi, 2010). While the precise etiology remains largely unknown, recent genetic and immunological studies have highlighted the critical roles of both innate and adaptive immune systems, particularly the activation of T helper 1 (Th1) and T helper 17 (Th17) cells, and the dysregulation of antigen-presenting cells (Takeuchi et al., 2021; Rosenbaum et al., 2019). Genetic predisposition is also a significant factor in disease development; for example, human leukocyte antigen B27 (HLA-B27) is strongly associated with acute anterior uveitis, and human leukocyte antigen A29 (HLA-A29) with birdshot chorioretinopathy (Huang and Brown, 2022; Kuiper et al., 2015). Despite advances in biological therapies targeting specific cytokines such as tumor necrosis factor-alpha (TNF-α) and interferons (Touhami et al., 2019), the clinical management of NIU remains challenging due to its unpredictable clinical course and high relapse rates (Rojas-Carabali et al., 2025). In addition, the development and validation of sensitive and reliable biomarkers that reflect disease activity and treatment response in uveitis remain significant unmet needs (Denniston et al., 2017).

Dendritic cells (DCs), acting as professional antigen-presenting cells, bridge the gap between innate and adaptive immunity and are increasingly recognized as playing a role in the pathogenesis of autoimmune uveitis (O'Rourke et al., 2018; Zhao and Yu, 2024). Studies in animal models have demonstrated that local DC activation can directly alter the pathogenesis of retinal autoimmune disease (Heuss et al., 2012). In particular, recent transcriptome network analyses have identified a unique CX3CR1-positive type 3 dendritic cell (DC3) subtype that is significantly diminished in the peripheral blood of NIU patients and exhibits a pro-inflammatory gene expression profile (Hiddingh et al., 2023). Furthermore, innate immune receptors, such as Toll-like receptor 7 (TLR7), and its regulatory molecule CD180, have been shown to modulate DC activation and subsequent inflammatory cascades in autoimmune conditions (Yang et al., 2018). In experimental autoimmune uveitis models, suppression of TLR7 expression in the retinal pigment epithelium has been shown to reduce disease severity, supporting the direct role of TLR7 in uveitis pathogenesis (Lo et al., 2022). However, the comprehensive transcriptomic appearance of these specific DC subtypes in NIU and their potential as diagnostic biomarkers using advanced computational methods has not yet been fully investigated.

In recent years, high-throughput transcriptomic technologies, from microarray-based gene expression profiling (Mesko et al., 2011) to RNA sequencing (RNA-seq) (Ma et al., 2022), have made significant contributions to elucidating molecular signatures in autoimmune diseases. In the context of uveitis, peripheral blood transcriptomics has shown promise for refining the diagnosis of idiopathic cases and identifying common molecular pathways across uveitis subtypes (Rosenbaum et al., 2021a; Rosenbaum et al., 2021b; Li et al., 2008). In particular, Rosenbaum et al. (2021b) demonstrated that peripheral blood gene expression profiling can serve as a potential adjunct tool for accurate differential diagnosis of uveitis etiology. A recent integrative immunology study highlighted the systemic nature of the disease by identifying distinct interferome signatures in the blood transcriptome of NIU patients (Munhoz et al., 2025). Simultaneously, the application of machine learning (ML) in ophthalmology and rheumatology has demonstrated exceptional capabilities for processing high-dimensional clinical and multi-omics data to classify diseases and predict outcomes (Jacquot et al., 2023; Jacquot et al., 2025; Li et al., 2025). More broadly, within a systems-biology and precision medicine framework, artificial intelligence (AI)-driven integration of multi-omics and multimodal data has become essential for uncovering complex disease mechanisms and guiding personalized therapeutic strategies (Liu, 2026). Furthermore, comprehensive analyses of molecular determinants via multi-omics integration have proven highly effective in identifying systems-level biomarkers across various complex diseases, including oncology and autoimmunity (Ubaid et al., 2025). For example, ML algorithms have been successfully used to develop classification criteria for uveitis (The Standardization of Uveitis Nomenclature SUN Working Group, 2021) and to identify immunological regulators linking ankylosing spondylitis and uveitis (Cai et al., 2025). Similarly, an interpretable ML pipeline trained on blood transcriptomic data has yielded successful results in phenotype prediction for heterogeneous autoimmune diseases, such as systemic lupus erythematosus (Leventhal et al., 2023).

Despite these advances, a significant limitation of traditional ML models in clinical transcriptomics is their *black box* structure, which obscures the biological interpretability of predictions (Lalman et al., 2025). Explainable Artificial Intelligence (XAI), particularly the Shapley Additive Explanations (SHAP) framework (Lundberg and Lee, 2017), has emerged as a powerful tool to overcome this obstacle by quantifying each feature’s (e.g., gene) contribution to the model output. AI has recently enabled precise diagnosis and biomarker discovery in complex ocular diseases, such as diabetic retinopathy (Yagin et al., 2023). The use of penalized models, such as penalized regression, for biomarker selection in gene expression data is considered an effective strategy in preventing overfitting in high-dimensional data (Tibshirani, 1996; Zou and Hastie, 2005). Furthermore, integrating data from multiple independent cohorts often introduces cross-platform batch effects that require rigorous correction to ensure the generalizability of ML models (Kotlov et al., 2024; Foltz et al., 2023; Borisov and Buzdin, 2022).

To address these gaps, this study aims to identify robust, biologically interpretable transcriptomic biomarkers for NIU by applying an explainable machine-learning framework to CD1c+ conventional dendritic cell type 2 (cDC2) bulk RNA-seq data. A robust and interpretable L2-regulated logistic regression model was developed and validated by integrating two independent cohorts (GSE194060 and GSE195501) using rigorous batch-effect correction and feature selection. Furthermore, the critical roles of CD180, TLR7, and chemokine receptor/Toll-like receptor signaling pathways in the pathogenesis of non-infectious uveitis were highlighted using SHAP analysis and pathway enrichment to elucidate the underlying molecular mechanisms.

The remainder of this manuscript is organized as follows: Section 2 details the data collection, preprocessing, batch correction, feature selection, and model development strategy; Section 3 presents the classification performance, overfitting analysis, subgroup evaluation, explainable AI (SHAP) results, and pathway enrichment findings; Section 4 discusses the results within the context of the current literature, including the clinical relevance of the 20-gene signature and study limitations; and Section 5 concludes the study.

## 2 Methods

### 2.1 Data acquisition and study design

In this study, two independent, publicly available bulk RNA-sequencing (RNA-seq) datasets were obtained from the National Center for Biotechnology Information (NCBI) Gene Expression Omnibus (GEO) database to investigate NIU pathogenesis at the transcriptomic level and discover diagnostic biomarkers. The datasets included in the study are studies GSE194060 (Discovery Cohort) and GSE195501 (Replication Cohort), derived from the original study conducted by Hiddingh et al. (2023). Both datasets contain transcriptomic profiles of CD1c+ conventional dendritic cell type 2 (cDC2) populations isolated from peripheral blood mononuclear cells (PBMC). The demographic and clinical characteristics of the study population are summarized in Table 1.

**TABLE 1:** Demographic and clinical characteristics of the datasets included in the study.

Characteristic	GSE194060 (discovery cohort)	GSE195501 (replication cohort)	Total
Total samples	42	36	78
NIU patients	28	23	51
HLA-B27+ acute anterior uveitis	9	10	19
HLA-A29+ birdshot uveitis	10	8	18
Idiopathic intermediate uveitis	9	5	14
Healthy controls	14	13	27
Cell type	CD1c+ cDC2	CD1c+ cDC2	-
Sequencing platform	Illumina NovaSeq 6000	Illumina NextSeq 550	-
Tissue source	PBMC	PBMC	

As shown in Table 1, the study population comprises 78 samples. The GSE194060 dataset contains 42 samples in total, including 28 NIU patients (9 with HLA-B27+ acute anterior uveitis, 10 with HLA-A29+ birdshot uveitis, and 9 with idiopathic intermediate uveitis) and 14 healthy controls. This dataset was sequenced using the Illumina NovaSeq 6000 platform. The GSE195501 dataset, used for independent validation, contains 36 samples in total, including 23 NIU patients (10 HLA-B27+ acute anterior uveitis, 8 HLA-A29+ birdshot uveitis, 5 idiopathic intermediate uveitis) and 13 healthy controls. This second cohort was sequenced using the Illumina NextSeq 550 platform. Using two different sequencing platforms provides a robust *real-world* test of the platform-independent generalizability of the machine learning model being developed.

**FIGURE 1:**
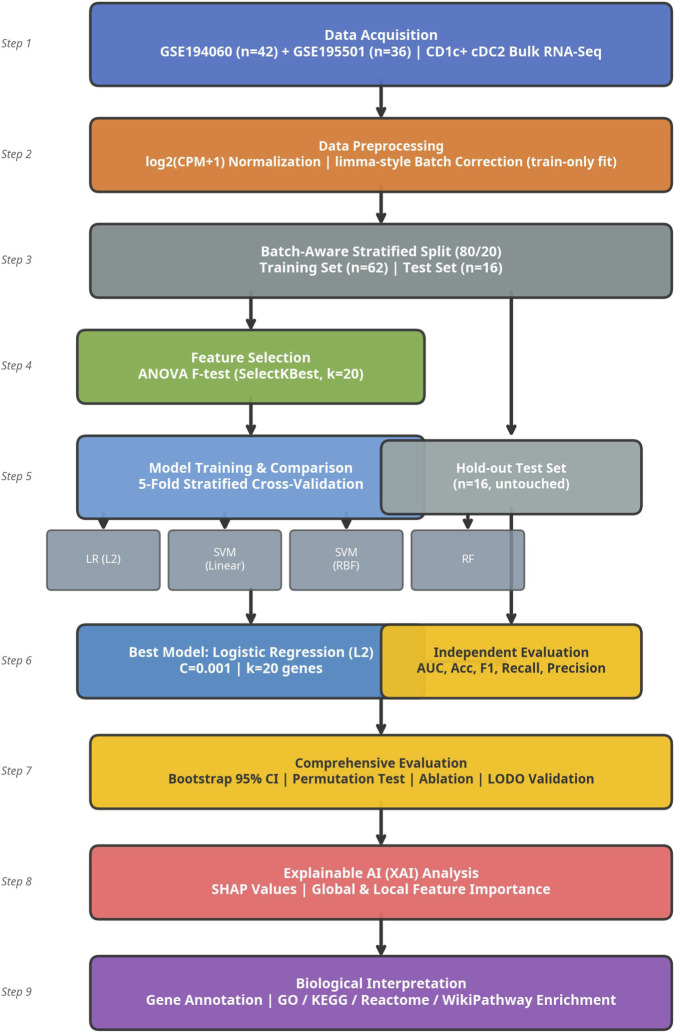
Overall workflow and machine learning architecture of the study.

The overall workflow of the study and the proposed machine learning architecture are shown in Figure 1.

As shown in Figure 1, the analysis pipeline consists of nine core steps: data acquisition, preprocessing, feature selection, model training and comparison, independent validation, XAI analysis, and biological interpretation.

### 2.2 Data preprocessing and normalization

Raw gene expression count matrices from both datasets were processed using Python 3.11. In the first stage, individual sample-level count files for the GSE194060 and GSE195501 datasets were downloaded from the GEO database, and a single combined gene-sample matrix was created for each dataset. Both matrices were matched using common ENSEMBL gene identifiers to obtain a combined expression matrix containing 65,217 genes.

In the normalization phase, the raw count data were first subjected to a Counts Per Million (CPM) transformation, followed by a log2(CPM+1) transformation. This transformation approximates gene expression distributions to a nearly normal distribution, meeting the assumptions of statistical analysis and reducing the dominant effect of highly expressed genes (Borisov and Buzdin, 2022). A +1 pseudocount was added to prevent zero expression values ​​from becoming undefined in the logarithmic transformation.

Systematic technical variations (batch effects) arising from two different sequencing platforms (NovaSeq 6000 and NextSeq 550) can cause machine learning models to learn technical artifacts rather than biological signals. To address this problem, a limma-style Ordinary Least Squares (OLS) regression-based batch correction method was applied (Borisov and Buzdin, 2022; Foltz et al., 2023). In this method, the expression level for each gene is modeled as the dependent variable, and the batch (data set source) is the independent variable, and the predicted batch effect is subtracted from each gene. Mathematically, the correction applied to each gene g in sample i was defined as follows:

$$x\_\left(corrected\right)\left(g,i\right)=x\_\left(original\right)\left(g,i\right)-\beta \_\left(batch\right)\left(g\right)\cdot batch\;\left(i\right)$$ (1)

As shown in Equation 1, $x_{\text{original}}\left(g,i\right)$ denotes the normalized expression value, $\beta_{\text{batch}}\left(g\right)$ represents the batch coefficient estimated by OLS regression, and $\text{batch}\left(i\right)$ indicates the dataset membership of the sample (0 or 1). To prevent data leakage, the batch-correction model was learned exclusively on the training set, and the estimated coefficients were subsequently applied to the test set. Further details are provided in Section 2.3.

In the final stage of preprocessing, a two-stage unsupervised gene filtering strategy was applied to reduce dimensionality while preserving biologically significant signals. In the first step, genes with negligible expression were removed by retaining only those expressed above 1 CPM (counts per million) in at least 10% of training samples. In the second step, the remaining genes were ranked by their expression variance across training samples, and the top 50% highest-variance genes were retained, yielding 8,767 genes for downstream analysis. Crucially, both filtering steps were computed exclusively on the training set (n = 62) and did not utilize disease labels, ensuring that this unsupervised preprocessing step introduced no information leakage. The same gene filter was then applied to the test set. A supplementary robustness analysis confirmed that performing this variance filtering inside the cross-validation loop produced virtually identical results in terms of cross-validation area under the curve (CV AUC: 0.910 vs. 0.916; test AUC: 0.855 vs. 0.855; 100% gene overlap), validating that this unsupervised filtering step does not bias model performance estimates.

### 2.3 Data partitioning and leakage prevention

To accurately assess the generalizability of machine learning models and prevent overfitting, the combined dataset was split into two parts: 80% training (n = 62) and 20% independent testing (n = 16). This splitting was performed using a batch-aware stratified split, which ensured that both disease/healthy control class ratios and dataset sources (batches) were preserved in both sets. A combination of disease label and dataset source (label + batch) was used as the stratification variable, thus algorithmically guaranteeing balanced representation of both platforms (GSE194060 and GSE195501) in both the training and test sets. The training set comprised 40 patients and 22 healthy controls, while the test set comprised 11 patients and 5 healthy controls.

A strict methodological approach was adopted to definitively prevent data leakage. The batch correction model (OLS regression coefficients defined in Section 2.2) was learned only on the training set, and the resulting coefficients were applied to the test set. Similarly, variance-based gene filtering thresholds were calculated only from the training set and transferred to the test set using the same gene filter. All data transformation operations, such as feature selection and scaling, were computed using only the training set, and the resulting parameters were directly applied to the test set. For this purpose, the Pipeline architecture of the Scikit-learn library was used; the feature selection (SelectKBest), standardization (StandardScaler), and classifier (Logistic Regression) steps were combined in a single pipeline. This structure, by requiring feature selection to be redone at each training layer (fold) during 5-fold cross-validation, prevented information leakage to the validation layer and ensured an unbiased estimate of the model’s real-world performance.

The supervised feature selection step (analysis of variance (ANOVA) F-test via SelectKBest) was embedded within the scikit-learn Pipeline architecture together with StandardScaler and the classifier. This design ensures that feature selection is automatically re-computed at each fold during 5-fold cross-validation, preventing any information from the validation fold from influencing gene selection. A feature stability analysis across CV folds confirmed that 5 core genes were consistently selected in all 5 folds, and 14 of the 20 genes appeared in at least 3 of 5 folds (mean Jaccard similarity: 0.336), demonstrating moderate selection stability.

### 2.4 Feature selection and machine learning pipeline

To prevent overfitting in high-dimensional transcriptomic data (n = 8,815 genes) and to identify the most biologically significant biomarkers, a statistical feature selection method was applied. Feature selection was performed only on the training set (n = 62) using the SelectKBest function from the Scikit-learn library and the ANOVA F-test (f_classif). This method selects the genes showing the most statistically significant variance between disease and healthy control groups.

To determine the optimal number of genes (k) and machine-learning hyperparameters while preventing optimism bias, a nested cross-validation framework was employed. The nested architecture consisted of an outer 5-fold StratifiedKFold loop for unbiased performance estimation and an inner 5-fold StratifiedKFold loop for hyperparameter tuning via grid search. Candidate gene numbers were defined as k ∈ {5, 10, 15, 20, 25, 30, 40, 50, 75, 100}, and the inverse regularization parameter C ∈ {0.001, 0.005, 0.01, 0.05, 0.1, 0.5, 1.0} values were tested for the L2-regularized Logistic Regression model. In each outer fold, the inner loop selected the best hyperparameters based solely on inner cross-validation AUC, which were then evaluated on the held-out outer fold. The independent test set (n = 16) was strictly isolated and never used during this nested optimization process. The most frequently selected optimal feature subset size across the nested folds was k = 20, and the final model was trained using the hyperparameters k = 20 and C = 0.001 on the full training set.

The final machine learning pipeline consists of three sequential steps: (1) selecting the top 20 genes using ANOVA F-test, (2) converting the selected features to a standard normal distribution (mean = 0, standard deviation = 1) using StandardScaler, and (3) an L2-Logistic Regression classifier using the limited-memory Broyden–Fletcher–Goldfarb–Shanno (LBFGS) solver configured with the class_weight = *balanced* parameter to correct class imbalance. To evaluate the comparative performance of the model, linear support vector machine (linear SVM), Support Vector Machines with radial basis function kernels (SVM-RBF), and Random Forest algorithms were also trained and tested using the same pipeline architecture.

### 2.5 Performance evaluation and statistical validation

The classification performance of the developed machine learning model was evaluated using standard metrics on an independent test set. The area under the receiver operating characteristic curve (AUC) was used as the primary metric to measure the model’s discriminative power. Additionally, the model’s overall accuracy, F1 score, recall (sensitivity), and precision were calculated. To assess the potential effects of class imbalance, the model’s performance was further examined using subgroup analyses stratified by uveitis subtype (HLA-B27+ acute anterior uveitis (AAU), HLA-A29+ Birdshot, Idiopathic Intermediate) and dataset source (GSE194060, GSE195501).

Two different approaches were adopted to verify the statistical reliability of the obtained performance metrics. First, 95% confidence intervals (CIs) were calculated for all metrics by applying a bootstrap method with 1,000 iterations on the test set (Efron and Tibshirani, 1993). This method provides a robust statistical basis for estimating the population-level variance in model performance when sample sizes are limited.

To evaluate the model’s cross-platform generalization capacity, Leave-One-Dataset-Out (LODO) validation was performed. In this approach, the hyperparameters of the main model (k = 20, C = 0.001) were kept constant, and two separate validations were performed: in the first round, the model was trained only on GSE195501 and tested on GSE194060; in the second round, it was trained only on GSE194060 and tested on GSE195501. Thus, each dataset was used as a completely independent external validation cohort that the model had never seen before. This analysis provides a robust external validation framework for assessing the model’s generalizability across different sequencing platforms.

In addition, a permutation test was conducted to determine whether the model’s high performance was due to random chance (Ojala and Garriga, 2010). In this test, the disease labels in the training set were shuffled randomly, the model was retrained 200 times, and cross-validation AUC values were recorded in each iteration. An empirical p-value was calculated by evaluating the position of the actual model’s AUC score within the AUC distribution of the models trained with random labels. The statistical significance level was accepted as p < 0.05. All statistical analyses and performance evaluations were performed using the Scikit-learn library in the Python (v3.11) environment.

### 2.6 XAI analysis

To overcome the *black-box* nature of machine learning models and ensure the biological interpretability of predictions, XAI techniques were used. In this context, SHAP framework, based on game theory and fairly quantifying the marginal contribution of each trait (gene) to the model output, was applied (Lundberg and Lee, 2017).

Given the linear nature of the L2-regulated Logistic Regression model, the LinearExplainer module was chosen for the analyses. SHAP values were calculated using standardized training and test sets, and the directional effect (positive or negative) and effect size of each gene on predicting disease (NIU) or healthy control class were determined. To rank the overall importance of the genes, the mean absolute SHAP value of all samples was calculated.

In addition to the SHAP analysis, a permutation feature importance analysis was performed to verify the model’s feature dependence. In this method, the expression values of each gene in the test set were randomly shuffled, and the decrease in the model’s AUC score was measured. This process was applied with 30 repetitions (n_repeats = 30) to ensure the statistical stability of the results. By combining the findings from both XAI approaches, the biomarker candidates with the highest diagnostic value in the pathogenesis of non-infectious uveitis were identified.

### 2.7 Gene annotation and pathway enrichment analysis

A comprehensive gene annotation and pathway enrichment analysis was performed to elucidate the biological functions and pathogenic roles of 20 genes selected by the machine learning model. In the first stage, the ENSEMBL gene identifiers used by the model were converted into official gene symbols and full gene names by querying the NCBI Entrez database via the Python-based mygene library (v3.2.2) (Xin et al., 2016).

To determine the roles of annotated genes in cellular processes and signaling pathways, overrepresentation analysis was performed using the gseapy library (v1.1.1) via the Enrichr application programming interface (API) (Chen et al., 2013; Kuleshov et al., 2016). The analyses were carried out using the Gene Ontology (GO) Biological Process, GO Molecular Function, GO Cellular Component, WikiPathways, and BioPlanet libraries.

Enrichment analysis was performed on all 20 selected genes. According to the SHAP analysis, all 20 genes were disease-associated, thereby eliminating the need for a separate subset analysis. Statistical significance was assessed using the Benjamini-Hochberg method for multiple testing correction, and terms with an adjusted p-value < 0.05 were considered statistically significant.

## 3 Results

### 3.1 Cross-validation and machine learning model performance

Four machine learning algorithms were trained and evaluated on the training set (n = 62) using an independent test set (n = 16). For the final L2-regularized Logistic Regression model, a nested cross-validation framework (outer 5-fold for unbiased performance estimation, inner 5-fold for hyperparameter tuning) was employed, while the remaining models were evaluated using standard 5-fold cross-validation. In all cases, 20 genes were selected via ANOVA F-test, model selection was based solely on cross-validation performance, and the independent test set was never used during hyperparameter optimization. Cross-validation and independent test set performance metrics for all models are presented in Table 2.

**TABLE 2:** Machine learning model performance comparison with nested and standard cross-validation.

Model	CV AUC (mean ± SD)	Nested CV AUC (mean ± SD)	Train AUC	Test AUC	Test accuracy	Test F1	Test recall	Train-test gap
Logistic regression (L2)	0.916 ± 0.102	0.863 ± 0.126	0.960	0.855	0.812	0.870	0.909	0.105
SVM (linear)	0.916 ± 0.102	-	0.959	0.855	0.750	0.846	1.000	0.104
Random forest	0.845 ± 0.096	-	1.000	0.909	0.750	0.833	0.909	0.091
SVM (RBF)	0.835 ± 0.139	-	0.942	0.164	0.688	0.815	1.000	0.778

As shown in Table 2, the L2-regularized Logistic Regression (L2, C = 0.001) model, evaluated via a nested cross-validation framework, achieved an unbiased mean AUC of 0.863 ± 0.126, yielding an AUC of 0.855, 81.2% accuracy, an F1 score of 0.870, and a sensitivity of 90.9% on the independent test set. The close alignment between the unbiased nested CV estimate (0.863) and the independent test set performance (0.855) confirms that the model possesses robust generalization capacity and is not severely overfitting. A comparison with standard non-nested cross-validation (AUC = 0.916) revealed a modest optimism bias of 0.053, which is expected for small biomedical datasets and underscores the importance of the nested framework for accurate performance estimation in high-dimensional transcriptomic data. Notably, the optimal feature subset size (k) selected by the inner loop varied across outer folds (k = 10, 50, 100, 20, 20), reflecting hyperparameter instability inherent to small-sample high-dimensional settings where multiple gene subsets achieve similar discriminative performance. Despite this variability, k = 20 emerged as the most frequently selected value (consensus choice), and the consistent test set performance (AUC = 0.855) confirms that the final model generalizes well regardless of this inner-loop instability. The SVM (RBF) model showed a significant performance drop on the test set (Test AUC: 0.164), indicating that the nonlinear kernel function is not suitable for this data structure. Although the Random Forest model showed high performance on test AUC (0.909), the training AUC of 1.000 and the large train-test gap (0.091) indicate that this model is prone to overfitting. Based on these results, the L2-regularized logistic regression (LR-L2), which offers the most balanced generalization capacity, was selected as the final classifier.

### 3.2 Overfitting analysis and statistical validation

To verify the model’s generalizability and statistical reliability, hyperparameter optimization, feature ablation, permutation tests, and bootstrap analyses were performed. The best-performing combinations for different feature numbers (k) and regularization coefficients (C) obtained during the optimization process are summarized in Table 3.

**TABLE 3:** Top 5 hyperparameter combinations from grid search.

Features (k)	Regularization (C)	CV AUC	Train AUC	Overfit gap
30	0.001	0.917	0.969	0.052
20	0.001	0.916	0.960	0.044
30	0.005	0.916	0.972	0.055
20	0.005	0.916	0.961	0.045
100	0.050	0.912	0.996	0.084

Selected model: k = 20, C = 0.001 and CV AUC, values in this table reflect standard (non-nested) cross-validation scores used during the grid search process. The unbiased performance estimate from nested cross-validation is reported in Table 2.

As shown in Table 3, while the combination of k = 30 and C = 0.001 yielded the marginally highest CV AUC score (0.917), the combination of k = 20 and C = 0.001 exhibited nearly equivalent performance (CV AUC = 0.916) while keeping the performance difference between training and test sets (overfit gap = 0.044) lower. Therefore, the k = 20 model was chosen as the final model to minimize overfitting and obtain a more interpretable, parsimonious biomarker profile.

**FIGURE 2:**
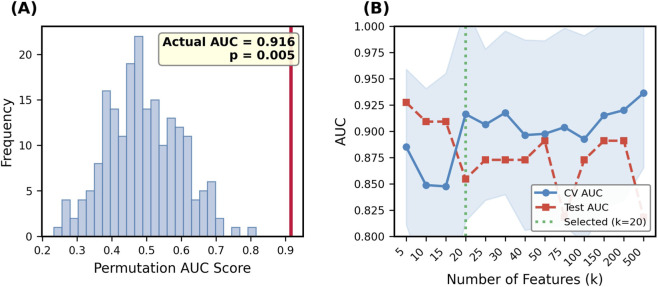
Statistical validation and feature selection analysis. **(A)** Permutation test distribution (n = 200); red line indicates actual model AUC (p = 0.005). **(B)** Exploratory feature ablation analysis showing performance trends across different feature subset sizes.

The results of the permutation test, conducted to prove that the model’s success was not due to chance, and the exploratory ablation analysis, which shows the effect of feature number on performance, are presented in Figure 2.

In the permutation test (A) presented in Figure 2, the standard CV AUC score of the actual model (0.916) was statistically significantly higher than the average AUC score of models trained with random labels (p = 0.005). In the exploratory feature ablation analysis (B), the change in model performance across different feature numbers was examined, and it was found that the 20-gene subset had the lowest overfit gap (overfit = 0.044) among all tested configurations and provided the best balance between high CV performance (standard CV AUC = 0.916) and generalization capacity.

Finally, 95% confidence intervals (CIs) were calculated using the 1,000-iteration bootstrap method to assess the model’s predictive reliability on the independent test set, and the results are presented in Table 4.

**TABLE 4:** Bootstrap confidence intervals (95% CI) for model performance metrics on the independent test set (1,000 iterations).

Metric	Mean	95% CI lower	95% CI upper
AUC	0.850	0.625	1.000
Accuracy	0.808	0.625	1.000
F1-score	0.861	0.695	1.000
Recall	0.904	0.692	1.000
Precision	0.831	0.615	1.000

The confidence intervals shown in Table 4 confirm that the model’s predictions are stable across independent cohorts. While lower confidence bounds exceed chance level (0.625), the narrow margins and wide interval (0.375) reflect substantial uncertainty from small test set (n = 16). Accordingly, the reported point estimates should be interpreted as preliminary evidence rather than definitive performance benchmarks.

After correcting steps that could lead to data leakage, the model continued to exhibit statistically significant performance (CV AUC = 0.916, nested CV AUC = 0.863, Test AUC = 0.855) using a simpler 20-gene signature. Although a moderate performance gap (train-test gap = 0.105) remained between the training and test sets, the statistical significance of the permutation test (p = 0.005), the consistency of the LODO cross-platform validation results (Table 5), and the fact that the lower bounds of the bootstrap confidence intervals exceeded the chance level, did not reveal any evidence of severe overfitting in the model. However, given the limited sample size, the results should be interpreted with caution.

**TABLE 5:** Leave-One-Dataset-Out (LODO) cross-platform validation results with fixed hyperparameters (k = 20, C = 0.001).

Training cohort	Test cohort	Platform (train → test)	AUC	Accuracy	Recall	F1
GSE195501 (n = 36)	GSE194060 (n = 42)	NextSeq 550 → NovaSeq 6000	0.806	0.738	1.000	0.836
GSE194060 (n = 42)	GSE195501 (n = 36)	NovaSeq 6000 → NextSeq 550	0.833	0.806	0.739	0.829
Mean	-	-	0.819	0.772	0.870	0.833

### 3.3 Cross-platform external validation

To evaluate the model’s generalizability across independent sequencing platforms, Leave-One-Dataset-Out (LODO) cross-validation was performed using fixed hyperparameters (k = 20, C = 0.001) from the main model. The LODO results are presented in Table 5.

As shown in Table 5, when the model was trained exclusively on GSE195501 (NextSeq 550, n = 36) and tested on GSE194060 (NovaSeq 6000, n = 42), it achieved an AUC of 0.806 with perfect recall (1.000). In the reverse direction, training on GSE194060 and testing on GSE195501 yielded an AUC of 0.833 with 73.9% recall. The mean LODO AUC of 0.819 confirms that the model maintains robust classification performance even when trained and tested on entirely separate cohorts from different sequencing platforms, providing strong evidence of cross-platform generalizability.

### 3.4 Subgroup analysis and threshold evaluation

To assess the model’s stability across different clinical subtypes and data sources, subgroup analyses were performed on an independent test set. The results of the subgroup analyses are summarized in Table 6.

**TABLE 6:** Subgroup performance across clinical subtypes and dataset batches in the test set.

Subgroup	Category	n	Accuracy	Recall	F1-score
Disease subtype	HLA-B27+ AAU	2	1.000	1.000	1.000
	HLA-A29+ birdshot	4	0.750	0.750	0.857
	Idiopathic intermediate	5	1.000	1.000	1.000
	Healthy controls	5	0.600	-	-
Dataset batch	GSE194060	9	0.778	0.833	0.833
	GSE195501	7	0.857	1.000	0.909

HLA-B27+ AAU, subgroup (n = 2); insufficient for statistical inference.

As shown in Table 6, the model correctly identified 3 of 4 patients in the HLA-A29+ Birdshot Uveitis subtype (75% recall) and all patients in the Idiopathic Intermediate Uveitis subtype. It should be noted that the HLA-B27+ AAU subgroup contains only 2 samples, which is insufficient to draw any meaningful conclusions regarding subtype-specific performance; the 100% classification reported for this subgroup is therefore anecdotal and cannot be generalized. In the healthy control group (n = 5), the accuracy remained at 60%, with 2 out of 5 healthy individuals being mistakenly classified as patients. This indicates that the model shows stronger discrimination in disease classification, but performance in healthy controls should be interpreted cautiously due to the limited sample size.

Based on the dataset source, the model’s consistent performance in both the GSE194060 (Accuracy: 0.778, AUC: 0.889) and GSE195501 (Accuracy: 0.857, AUC: 0.800) cohorts supports the conclusion that the batch effect is largely controlled and that the model is robust against different sequencing platforms (NovaSeq vs. NextSeq).

In the threshold analysis, the default threshold of 0.50 provided the most balanced performance with 81.2% accuracy, 90.9% sensitivity, and an F1 score of 0.870. The applicability of alternative threshold values is limited due to the narrow range of prediction probabilities (0.49–0.53). However, due to the extremely limited sample sizes in subgroup analyses (n = 2–5), these results are inherently unstable and should be considered exploratory rather than confirmatory. Reliable subgroup-level conclusions require validation in substantially larger, prospectively collected cohorts.

### 3.5 XAI and biomarker identification

**FIGURE 3:**
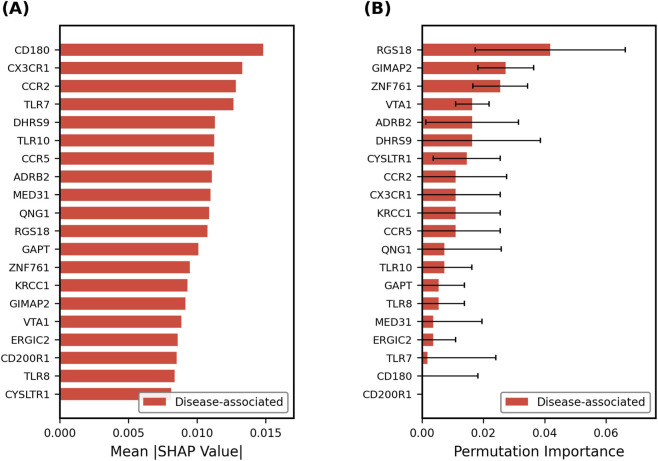
Feature importance analysis of the 20 selected genes. **(A)** Mean absolute SHAP values, all of which are disease-associated (red). **(B)** Permutation importance scores, confirming their critical role in model performance.

To improve transparency in the machine learning model’s decision-making process and identify potential biomarkers involved in the pathogenesis of non-infectious uveitis (NIU), SHAP and permutation-based feature importance analyses were performed. The genes that contributed most to the model’s predictions and their effects on disease are visualized in Figure 3.

According to the SHAP analysis (A) shown in Figure 3, the top five genes contributing most to the model’s predictive power were CD180, CX3CR1, CCR2, TLR7, and DHRS9, respectively. High expression levels of all 20 selected genes were found to strongly influence disease-up. Permutation significance analysis (B), conducted to confirm the SHAP findings, showed that the RGS18, GIMAP2, and ZNF761 genes had the greatest impact on model performance, confirming that both XAI methods highlighted genes associated with the immune system and inflammation.

Detailed information on the top 10 genes with the highest SHAP significance, along with model coefficients and effect directions, is summarized in Table 7.

**TABLE 7:** Top 10 genes identified by SHAP analysis with LR coefficients and association directions.

Gene symbol	ENSEMBL ID	SHAP importance	LR coefficient	Direction
CD180	ENSG00000134061	0.0148	+0.0184	Disease-associated
CX3CR1	ENSG00000168329	0.0133	+0.0172	Disease-associated
CCR2	ENSG00000121807	0.0128	+0.0160	Disease-associated
TLR7	ENSG00000196664	0.0127	+0.0178	Disease-associated
DHRS9	ENSG00000073737	0.0113	+0.0139	Disease-associated
TLR10	ENSG00000174123	0.0113	+0.0143	Disease-associated
CCR5	ENSG00000160791	0.0112	+0.0153	Disease-associated
ADRB2	ENSG00000169252	0.0111	+0.0143	Disease-associated
MED31	ENSG00000108590	0.0110	+0.0145	Disease-associated
QNG1	ENSG00000165118	0.0109	+0.0145	Disease-associated

As shown in Table 7, all of the top 10 genes are positively associated with disease pathogenesis. The high ranking of Toll-like receptor (TLR) family members CD180, TLR7, and TLR10, as well as chemokine receptors CX3CR1, CCR2, and CCR5, biologically supports the idea that innate immunity responses, leukocyte chemotaxis, and inflammatory signaling pathways play a critical role in the development of NIU.

### 3.6 Pathway enrichment analysis of selected biomarkers

A comprehensive pathway enrichment analysis was performed to elucidate the biological functions of 20 genes selected by the machine learning model. Since all selected genes were disease-up genes, the analysis was conducted on a single gene list. The analysis identified 237 statistically significant enriched terms based on the adjusted p-value threshold (p_adj < 0.05), and the most important biological processes and signaling pathways are summarized in Table 8.

**TABLE 8:** Top significant enriched pathways and biological processes for the 20 disease-associated genes (p_adj < 0.05).

Gene set library	Enriched term/Pathway	Adjusted p-value	Overlapping genes
GO molecular function	G protein-coupled chemoattractant receptor activity	8.0 × 10^−6^	CX3CR1, CCR5, CCR2
	Chemokine receptor activity	1.4 × 10^−5^	CX3CR1, CCR5, CCR2
	Cytokine receptor activity	6.3 × 10^−4^	CX3CR1, CCR5, CCR2
WikiPathways 2023	MYD88 distinct input output pathway	3.0 × 10^−5^	TLR8, TLR7, TLR10
	GPCRs, class A rhodopsin-like	1.0 × 10^−4^	CX3CR1, CYSLTR1, ADRB2, CCR5, CCR2
Reactome 2022	Toll-like receptor cascades	2.8 × 10^−4^	CD180, TLR8, TLR7, TLR10
	Chemokine receptors bind chemokines	2.9 × 10^−4^	CX3CR1, CCR5, CCR2
BioPlanet 2019	Innate immune system	5.2 × 10^−4^	CD180, TLR8, TLR7, TLR10, CCR2
GO biological process	Toll-like receptor signaling pathway	9.6 × 10^−4^	TLR8, TLR7, TLR10
	Positive regulation of type II interferon production	1.1 × 10^−3^	TLR8, TLR7, CCR2

As shown in Table 8, disease-associated genes are predominantly enriched in chemokine receptor activity, innate immunity, and inflammatory signaling pathways. Specifically, the *Toll-like receptor cascades* (p_adj = 2.8 × 10^−4^) and the *MYD88 signaling pathway* (p_adj = 3.0 × 10^−5^), spearheaded by the CD180, TLR7, TLR8, and TLR10 genes, highlight the critical role of innate immunity mechanisms in the pathogenesis of non-infectious uveitis. Furthermore, the presence of CX3CR1, CCR5, and CCR2 chemokine receptors, with the highest significance level (p_adj < 1.5 × 10^−5^), points to cellular-level triggers of leukocyte chemotaxis and intraocular inflammation. These findings confirm that the machine learning model not only delivers robust, interpretable classification performance but also selects clinically significant biomarkers consistent with the disease’s underlying biological mechanisms.

## 4 Discussion

This study aimed to classify the disease at the molecular level and identify potential biomarkers by applying explainable ML methods to transcriptomic data of CD1c+ conventional dendritic cell type 2 (cDC2) isolated from the peripheral blood of NIU patients. An L2-regulated logistic regression model, developed by integrating two independent cohorts (GSE194060 and GSE195501) and applying a strict data leakage prevention strategy, achieved high classification performance (nested CV AUC: 0.863, Test AUC: 0.855) using a 20-gene parsimonious signature. XAI analyses revealed that all 20 selected genes showed disease-up, and that CD180, TLR7, and chemokine receptors (CX3CR1, CCR2, CCR5) are computationally prioritized as key contributors in NIU pathogenesis.

Studies on the diagnosis and classification of uveitis in the literature generally focus on clinical features, demographic data, or ocular imaging methods (Jacquot et al., 2023; Jacquot et al., 2025; Rojas-Carabali et al., 2025). The integration of transcriptomic data with ML is a relatively new field. For example, Li et al. (2025) developed interpretable ML models based on clinical parameters to predict the development of anterior uveitis in patients with ankylosing spondylitis, while Cai et al. (2025) highlighted the immunological regulatory roles of HLX and SLC25A20 genes using multi-omics data. The study offers deeper insight into the cellular mechanisms of the disease by focusing directly on the gene expression profile of a specific immune cell subtype (CD1c+ cDC2) rather than on clinical data. In this respect, the study is methodologically similar to interpretable ML pipelines developed using blood transcriptomics in other autoimmune diseases, such as systemic lupus erythematosus (Leventhal et al., 2023; Ma et al., 2022), but represents one of the first interpretable ML applications specific to cDC2 cells in the NIU domain.

One of the strongest aspects of the study is the 20-gene parsimonious (simple) biomarker panel determined by feature ablation analysis. While selecting more genes in high-dimensional transcriptomic data (n = 8,815 genes) improves model training performance, the ablation analysis clearly showed that test performance decreased beyond 20 genes and that the risk of overfitting increased. This finding confirms the principle in machine learning that *more features are not always better*. The fact that all 20 selected genes are positively associated with disease-up demonstrates that the model eliminated noise genes and captured a biologically consistent signal. From a potential clinical translation perspective, once validated, the 20-gene panel could in principle be measured using targeted reverse transcription quantitative polymerase chain reaction (RT-qPCR) methods, which would be more cost-effective and accessible than full transcriptome RNA-seq profiling. However, it should be noted that the current workflow requires prior isolation of CD1c+ cDC2 cells from peripheral blood mononuclear cells (PBMCs), which is not a routine procedure in most ophthalmology clinical settings and represents a significant translational barrier that must be addressed in future studies. Furthermore, the presence of 237 significant terms (p_adj < 0.05) in pathway enrichment analysis, even with such a limited number of genes, indicates that the selected biomarkers were not random and strongly represent the core immunological mechanisms of the disease (Toll-like receptor cascades, MYD88 signaling pathway, chemokine receptor activity).

The most important biomarkers identified by the model, CD180 and TLR7, are in strong agreement with existing immunological findings in the literature. It has been previously shown that suppression of TLR7 in retinal pigment epithelium reduces the severity of experimental autoimmune uveitis (Lo et al., 2022). Similarly, CD180 has been shown to modulate TLR7-mediated activation of macrophages and dendritic cells (Yang et al., 2018). The fact that these two genes had the highest effector values in the SHAP analysis confirms the central role of innate immunity receptors in NIU pathogenesis. Additionally, consistent with the importance of the CX3CR1-positive DC3 subtype identified by Hiddingh et al. (2023), the developed model ranked chemokine receptors, including CX3CR1, CCR2, and CCR5, among the most critical biomarkers. From a machine learning perspective, this situation reaffirms the crucial role of leukocyte chemotaxis and tissue-specific cell migration in triggering intraocular inflammation (O'Rourke et al., 2018; Zhao and Yu, 2024). These findings align with the broader systems immunology framework, in which the identification of novel immune targets and signaling pathways, including TLR-mediated cascades, Janus kinase/signal transducer and activator of transcription (JAK/STAT), and mitogen-activated protein kinase (MAPK) pathways, has become central to understanding immune activation and developing precision immunotherapeutic strategies (Narote et al., 2025). Nevertheless, it is important to recognize that the biological interpretations derived from bulk transcriptomic data should be approached with caution, as traditional bulk analysis may mask cell-specific gene activities and the distinction between correlation and causation in gene expression requires careful consideration (Liu et al., 2024a).

While some of the key genes identified in our 20-gene signature, such as CX3CR1, CD180, and CCR2, were also reported in the original dataset publication by Hiddingh et al. (2023), the present study introduces several critical novelties to the literature. First, while the original study employed an unsupervised gene co-expression network analysis (WGCNA) to characterize the CX3CR1+ DC3 cellular subtype, our study utilizes a supervised, explainable machine learning framework to develop a predictive diagnostic model. Second, we distilled the high-dimensional transcriptomic data into a parsimonious 20-gene panel, transforming exploratory biological findings into a computationally validated biomarker signature that, pending further experimental validation and workflow simplification, could potentially be adapted for targeted RT-qPCR-based testing. Third, our XAI-driven approach identified and ranked several highly informative genes that were not highlighted in the original publication, including TLR7, TLR10, CCR5, DHRS9, RGS18, GIMAP2, and ZNF761. Finally, by conducting a comprehensive pathway enrichment analysis, we explicitly mapped these biomarkers to Toll-like receptor cascades and MYD88-mediated signaling, providing a deeper, systems-level understanding of the innate immune mechanisms driving non-infectious uveitis.

It is important to acknowledge that several of the key genes identified in this study, including TLR7, CX3CR1, CCR2, and CCR5, are well-established components of innate immune activation and inflammatory signaling pathways. The present study does not claim to have discovered novel gene functions; rather, its primary contribution lies in the computational prioritization and integration of these genes into a unified, parsimonious 20-gene diagnostic signature derived specifically from CD1c+ cDC2 transcriptomic data. While individual gene-pathway associations are known from prior immunological research, the demonstration that these genes collectively form a robust, cross-platform validated classification model (nested CV AUC = 0.863, independent test AUC = 0.855) for NIU represents a translational advance from descriptive immunology toward computationally prioritized candidate biomarker panels. Furthermore, several genes within the 20-gene signature, notably DHRS9, RGS18, GIMAP2, and ZNF761, have not been previously discussed in the context of NIU pathogenesis and warrant further functional investigation. The SHAP-based quantification of each gene’s directional contribution to disease classification provides an interpretive layer that extends beyond traditional differential expression analysis, offering mechanistic hypotheses regarding the relative importance of each pathway in cDC2-mediated NIU pathology.

Despite the study’s methodological strength and biological consistency, it has important limitations that warrant cautious interpretation of the results. First, the total sample size (n = 78) and especially the independent test set (n = 16) are small for a transcriptomic machine learning study, introducing inherent instability in performance estimates. This is reflected in the wide bootstrap confidence intervals (with upper bounds reaching 1.000) and the limited model performance (60% accuracy) in the healthy control subgroup. Furthermore, the feature stability analysis revealed that only 5 of the 20 selected genes were consistently chosen across all 5 cross-validation folds (mean Jaccard similarity: 0.336), indicating moderate selection instability. This is an expected consequence of the high-dimensional feature space (8,767 genes) relative to the small sample size (n = 62 training samples), where multiple correlated gene subsets can yield comparable classification performance. Importantly, despite this selection variability, the nested cross-validation AUC (0.863) and independent test set AUC (0.855) remained robust, suggesting that the selected genes capture overlapping biological signals rather than noise. Nevertheless, this instability underscores the need for validation in larger cohorts to identify the most reproducible subset of the 20-gene signature. Accordingly, the reported AUC values should be interpreted as promising preliminary estimates rather than definitive clinical performance metrics, and the findings require confirmation in larger, independent cohorts. Second, the data were retrospectively obtained from publicly available databases (GEO), and both cohorts were generated by the same research group at the same institution. Moreover, bulk transcriptomic repositories inherently carry both technical and biological biases that can influence downstream analyses. Technical biases arising from differences in sequencing platforms, library preparation protocols, and normalization methods, as well as biological biases stemming from sample heterogeneity and cellular composition variability, can affect the reliability of differential expression and pathway enrichment results derived from public datasets (Liu et al., 2024a; Liu et al., 2025b). Although batch correction was applied in this study using an OLS regression-based approach, residual platform-specific artifacts cannot be entirely excluded. Furthermore, as bulk RNA-seq captures averaged gene expression across all cells within the isolated CD1c+ cDC2 population, subtle transcriptomic heterogeneity within this cell subset may be masked, a limitation that could be addressed in future studies using single-cell RNA-seq (Liu et al., 2024a). While the LODO validation demonstrates robustness to sequencing platform differences (NovaSeq 6000 vs. NextSeq 550), it does not constitute a truly independent external validation equivalent to multicenter or geographically diverse cohorts, as potential confounders such as sample processing protocols, patient demographics, and laboratory-specific technical variability remain shared across both datasets. Therefore, the cross-platform generalizability demonstrated here should be considered a necessary but not sufficient condition for clinical translation, and definitive external validation requires future studies using cohorts collected independently at different centers and geographic regions. Third, the current analytical workflow requires isolation of CD1c+ cDC2 cells from PBMCs using fluorescence-activated cell sorting (FACS) or magnetic bead separation, followed by bulk RNA-seq profiling. These procedures are not routinely available in standard ophthalmology clinical settings, representing a significant translational barrier. Future translational efforts should explore whether the identified 20-gene signature can be reliably detected using more accessible platforms, such as targeted RT-qPCR panels applied directly to unsorted PBMCs or whole blood, which would substantially lower the barrier to clinical implementation. Fourth, the study focused only on CD1c+ cDC2 cells; integrating transcriptomic profiles of other immune cell populations, such as T cells or monocytes (Munhoz et al., 2025), could further enhance the model’s predictive power. Finally, and most importantly, this study remains an association-driven transcriptomic machine learning analysis without direct mechanistic or experimental confirmation. The identified biomarkers have not been validated through functional assays (e.g., gene knockdown or overexpression experiments), cytokine-level protein quantification, single-cell or spatial transcriptomics confirmation, RT-qPCR validation in an independent clinical cohort, or longitudinal disease activity analyses. Therefore, the biological interpretations derived from pathway enrichment and SHAP analysis should be considered as computationally generated hypotheses rather than experimentally confirmed mechanisms. A progressive validation framework, as exemplified by recent work on voltage-gated sodium channel (VGSC) biomarkers in glioma, demonstrates how computational biomarker discovery can be systematically strengthened through sequential experimental validation. In that paradigm, an initial comprehensive bioinformatics review (Liu et al., 2024c) was followed by data-driven prognostic biomarker identification (Liu et al., 2024b), and ultimately confirmed through functional cell biology experiments demonstrating that the β3 subunit modulates glioma cell motility independently of channel activity (Liu et al., 2025a). Adopting a similar stepwise approach, future studies should aim to validate the 20-gene NIU signature through RT-qPCR in independent prospective cohorts, followed by functional experiments targeting the top-ranked genes (CD180, TLR7, CX3CR1) in cDC2 cell culture models, and ultimately through longitudinal clinical studies correlating biomarker expression with disease activity and treatment response.

In conclusion, this study demonstrates that explainable machine learning is a powerful tool for elucidating the pathogenesis of non-infectious uveitis and discovering diagnostic biomarkers. The developed 20-gene parsimonious signature offers both high classification performance and accurately reflects the underlying Toll-like receptor and chemokine signaling pathways of the disease. Future studies should aim to validate this biomarker panel in larger clinical cohorts and to use it to develop novel targeted therapeutic strategies.

## 5 Conclusion

In this study, a parsimonious diagnostic biomarker signature comprising 20 genes was developed using explainable machine learning on bulk RNA-seq data from CD1c+ cDC2 cells from patients with non-infectious uveitis (NIU). The L2-regulated logistic regression model achieved an unbiased nested cross-validation AUC of 0.863, and 0.855 in the independent test set, and its statistical reliability was proven by permutation testing (p = 0.005) and LODO validation. SHAP analysis identified CD180, TLR7, CX3CR1, CCR2, and CCR5 as the most important biomarkers; pathway enrichment analysis confirmed that these genes were significantly enriched for Toll-like receptor cascades, the MYD88 signaling pathway, and chemokine receptor activity. The findings reveal the potential of XAI to elucidate disease mechanisms in transcriptomic data and identify candidate diagnostic biomarkers that warrant further clinical validation. Future studies are recommended to validate this 20-gene panel in independent prospective cohorts and with clinical methods such as RT-qPCR.

## Data Availability

Publicly available datasets were analyzed in this study. This data can be found here: The original RNA-seq datasets are available from the NCBI Gene Expression Omnibus (GEO): GSE194060 (https://www.ncbi.nlm.nih.gov/geo/query/acc.cgi?acc=GSE194060) and GSE195501 (https://www.ncbi.nlm.nih.gov/geo/query/acc.cgi?acc=GSE195501). All source code, trained models, and preprocessed datasets are available at https://github.com/yeldafrt/NIU-cDC2-Biomarkers.
